# Speech outcome following primary furlow palatoplasty with buccal myomucosal flap versus two flap palatoplasty in patients with cleft palate

**DOI:** 10.1007/s00784-025-06695-6

**Published:** 2025-12-20

**Authors:** Shaimaa Mohsen Refahee, Mamdouh Ahmed Aboulhassan, Iman Mohamed Elrouby, Mohamed Abd-El-Ghafour

**Affiliations:** 1 Oral and Maxillofacial Surgery Department, Faculty of Dentistry, Fayoum University, Fayoum, Egypt; 2 Plastic Section, General Surgery Department, Faculty of Medicine, Cairo University, Cairo, Egypt; 3 Phoniatrics, Phoniatrics Department, Hearing and Speech Institute, Cairo, Egypt; 4 Department of Orthodontics, Faculty of Dentistry, Cairo University, Cairo, Egypt

**Keywords:** Cleft palate, Two flap palatoplasty, Furlow palatoplasty with buccal myomucosal flap cleft palates, Velopharyngeal insufficiency

## Abstract

**Objective:**

The current study aimed to evaluate the speech outcome of primary Furlow palatoplasty with buccal myomucosal flap (FPBF) versus two flap palatoplasty (TFP) in patients with cleft palate.

**Materials and methods:**

Thirty patients with cleft palate were included in the current study. Fifteen patients received the primary FPBF as the surgical palatal repair technique, while the other 15 patients received the primary TFP as their cleft palate repair. All surgeries were done by a single surgeon at the age of 9–12 months. Speech assessment was done at the age of 4–8 years, including the amount of hypernasality, speech intelligibility, compensatory misarticulation, and nasopharyngoscopy VP valve competence.

**Results:**

Statistically significant improvements were detected in the 4 assessment methods while comparing primary FPBF versus the TFP groups’ scores.

**Conclusion:**

Primary FPBF might be able to improve the speech outcome in comparison to TFP in patients with cleft palate.

**Clinical significance:**

Primary FPBF improves the amount of hypernasality, speech intelligibility, compensatory misarticulation, and nasopharyngoscopy VP valve competence. Accordingly, it limits the need for further VP repair surgeries.

**Trial registration:**

Clinicaltrials.gov (NCT06856330).

**Supplementary Information:**

The online version contains supplementary material available at 10.1007/s00784-025-06695-6.

## Introduction

Cleft palate with or without cleft lip is the most frequent head and neck birth anomalies [1, 2]. It represents approximately 1 in 1500–2000 births [2]. The main aims of cleft palate repair surgery are restoring the palatal muscles, improving maxillary growth, preserving the function of the middle ear, and developing normal speech [3, 4].

For the development of normal speech, the palatal muscle must have sufficient length and function to articulate properly with the pharyngeal wall to produce a competent velopharyngeal (VP) mechanism [5]. About 20–40% of repaired cleft palate cases developed velopharyngeal insufficiency (VPI) [6]. This might be due to the inability of the surgical technique to reorient the palatini muscles properly, the contraction of the resultant surgical scar, and shorting of the palatal muscle. Subsequently, the VP contact failed to be established [7, 8]. The characteristic symptoms of VPI are nasal resonance, leakage of air from the nose during speech, and maladaptive articulation [9–11].

Several techniques are available for primary palatoplasty, such as the V-Y technique, two-flap palatoplasty (TFP), Furlow palatoplasty, and Furlow palatoplasty with buccal myomucosal flap (FPBF) [12–14]. All these techniques are with swinging efficiency in relation to speech development, maxillary growth, and incidence of fistula formation [15]. The primary two flap palatoplasty was the most preferred technique for a long time. It is a simple procedure that can be implemented by the less experienced cleft surgeons [16]. It is associated with a limited area of bare palatal bone, levator sling repair, and a lower incidence of fistula development [17]. On the other hand, it results in short, and improperly oriented palatini muscles. Moreover, the incision scar contraction affects the mobility of soft palate muscles and endanger the VP mechanism [17, 18]. According to Salyer et al. [19] about 8.92% of patients with cleft palate who were repaired with TFP have VPI symptoms. In addition, Richardson and Krishna [20] found in their study that there was about 14.8% of the operated cleft patients with TFP have a speech defect.

Nowaday, FBPF is one of the preferred primary palatoplasty techniques to be followed [13, 14]. It combines the advantages of Furlow palatoplasty and that of the vascularized buccal myomucosal flap. It enables cleft gap closure with minimal tension (regardless of its width) as the buccal flap augments the deficient palatal tissues and subsequently reduces scar formation. In addition, it provides proper palatal muscle reorientation, length, and function [14, 21, 22]. Aboulhassan et al. [13] documented that there is about a 40% increase in the palatal length gained by using primary FPBF. For these reasons, it is associated with a good VP function and good speech production. [14, 21, 22]

After searching the literature, there is no published studies reporting the effect of primary FPBF versus the TFP on speech development. Accordingly, the goal of the current study was to evaluate the speech outcome of primary FPBF versus TFP in patients with cleft palate.

## Materials and methods

### Study design and setting

This cohort study protocol was revised by the ethical committee of Fayoum university (EC 2511) and was prospectively listed on clinicaltrials.gov (NCT06856330). It adhered to Helsinki’s statement and conformed to STROBE outline [23, 24]. Speech evaluation for all participants was performed between March and June 2025 after the participants’ guardian approved and signed the declared consent.

### Participants

Thirty medically free children between the ages of 4–8 years were enrolled in the current study. All participants suffered from non-syndromic unilateral complete cleft palate that was primarily repaired either by FPBF or TFP at the age range of 9–12 months. All the surgeries were performed by a single certified surgeon. Exclusion of bilateral cleft palates, syndromic patients, secondary palatoplasty, and palatal fistula cases was a must. Speech assessment for all participants was performed by a single blinded well trained phoniatrician. Speech assessment was done through perceptual speech assessment and nasopharyngoscopy.

### Study size calculation

The hypernasality score data of Aboulhassan et al. [25] study were used to calculate the sample size by an independent t-test. This data was 1.4 ± 0.6. G Power software was used to conduct the sample size with an 80% power value, 1.16 effect size, and 0.05 significance level. About 26 patients should be included in this study, but this number was increased to 30 patients, 15 in each group to avoid the attrition bias.

### Surgical procedures

The surgical procedures were begun after local anesthesia with adrenalin1/200.000 was injected.

**Fig. 1:**
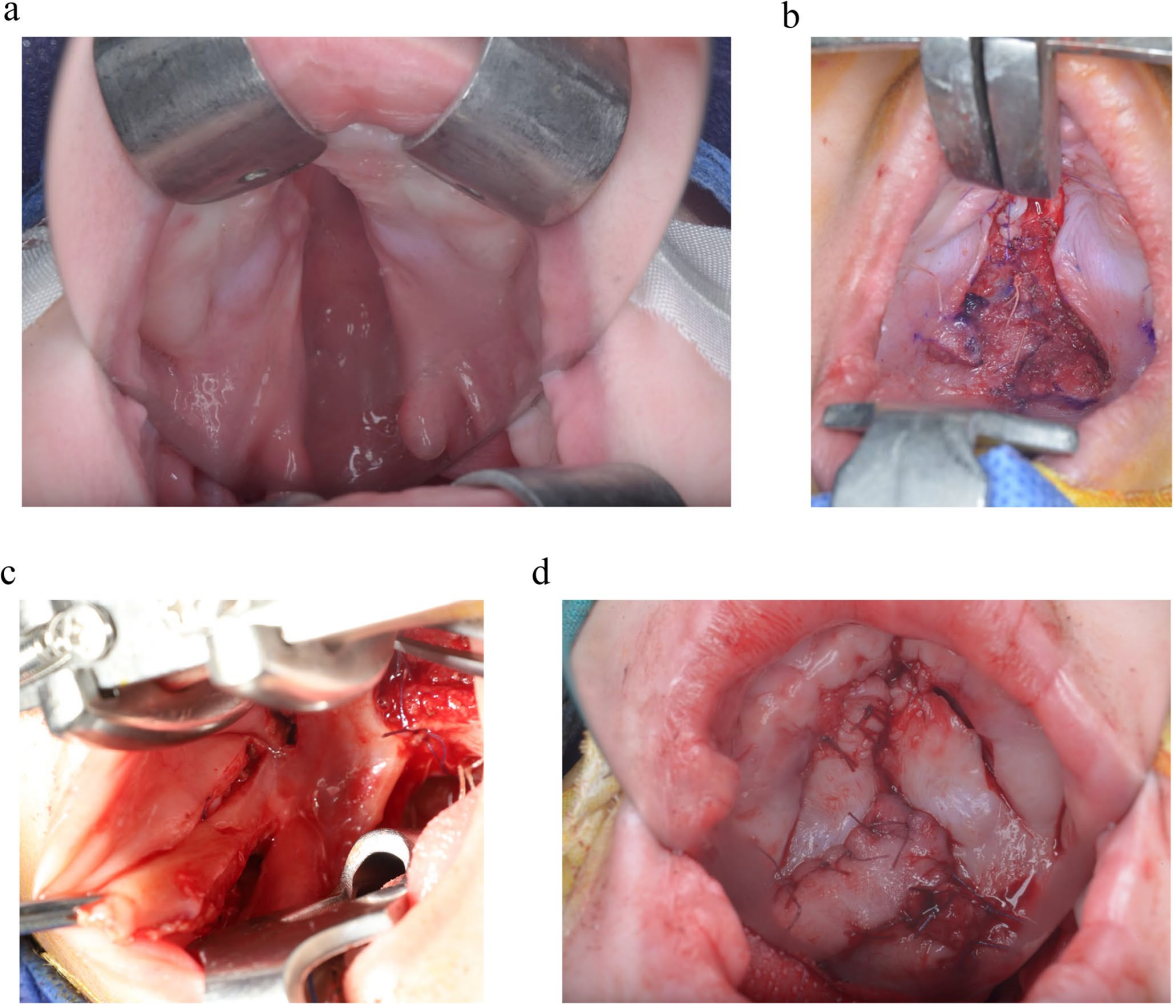
Surgical repair of cleft palate by primary FPBF. **a** Showing a preoperative unilateral complete cleft palate. **b** Showing a nasal layer closure after Z-plasty incision. **c** Showing a buccal myomucosal flap incision. **d** Showing a postoperative cleft palate closure by FPBF

Furlow palatoplasty with buccal myomucosal flap (FPBF) surgical steps (Fig. 1a,b,c,d):[13]

Z-plasty design was outlined on the palatal mucosa by methylene blue. On the left side, a posteriorly based myomucosal triangle (palatal mucosa and levator muscle) with an angle of approximately 60–80 degrees was elevated. A second anteriorly based mucosal triangle (nasal mucosa only) was incised the halfway between the uvula and the hard palate and extended laterally to Hamulus. On the right side, an anteriorly based oral mucosal 90-degree angle flap was created. A second posteriorly based myomucosal flap (nasal mucosa and levator muscle) was elevated. After all flaps had been elevated, nasal, muscle, and oral mucosal layers were interdigitated and closed with minimal overlapping of the palatine muscle.

A buccal myomucosal flap measuring 3.5 to 5.0 cm in length and 1.2 to 1.5 cm in width was designed and incised below the parotid duct. The elevated flap containing the bulk of muscle fibers and the remaining thin fiber was left to preserve the cheek fat. The flap was rotated and sutured to the palatal mucosa to substitute the deficient area of it. Finally, the inner side of check was sutured.

**Fig. 2:**
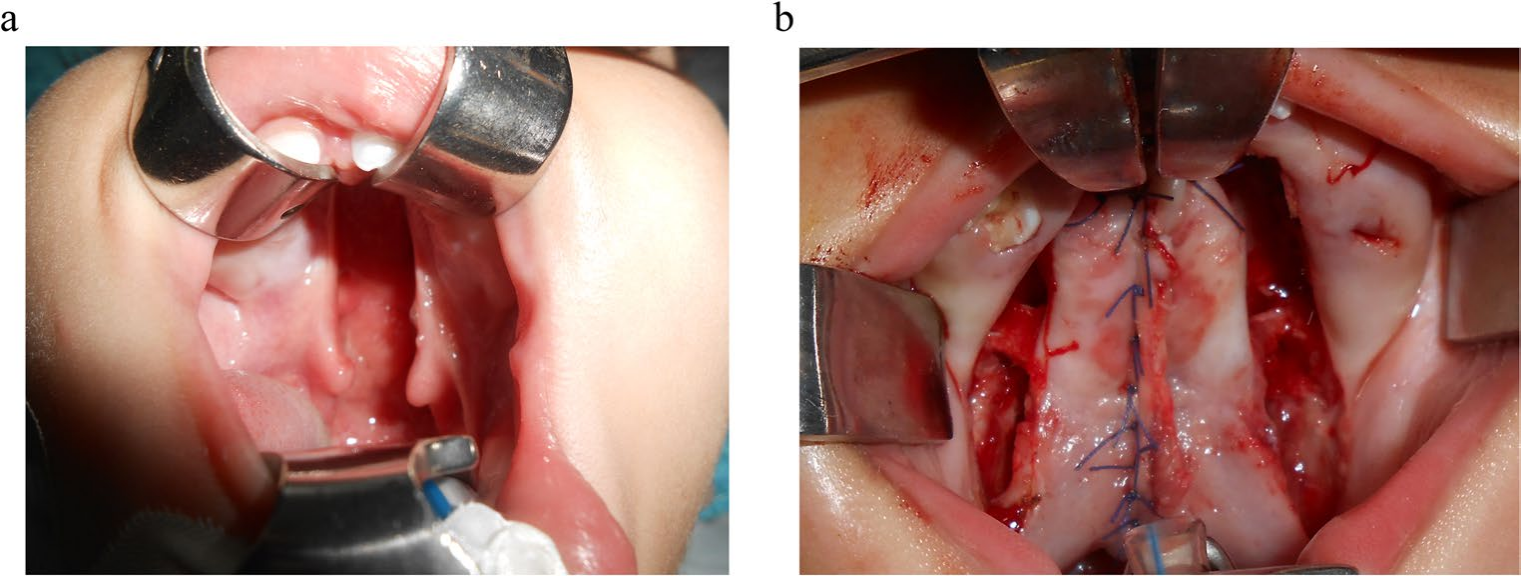
Surgical repair of cleft palate by primary TFP. **a** Showing a preoperative unilateral complete cleft palate. **b ** Showing a postoperative cleft palate closure by TFP

Two flap palatoplasty surgical steps (Fig. 2a,b) [26]

A bilateral tongue like palatal flaps were incised and elevated from the incisive foramen anteriorly to the hamulus posteriorly medial to the teeth and extended around the cleft area. Palatal muscles were detached from the palatal shelves and separated it from the nsal mucosa. The nasal layer was the firstly closed, followed by the muscle and oral mucosal layers. In case of the presence of a bare area of bone, it was left to heal by secondary intention. 

## Data measurements (speech evaluation)

### Perceptual speech evaluation

It is a subjective method to identify the speech abnormalities by an expert phoniatrician. It was completed by asking the child to repeat various syllables, words, phrases, and numbers (1 to 10). These records were collected to determine the severity of speech abnormality in the form of hypernasality, intelligibility, and compensatory speech.

Hypernasality and speech intelligibility were rated on 4-point scales (0–3) as (0) reflected no hypernasality/normal intelligibility, where (3) reflected severe hypernasality/severe intelligibility impairment. Compensatory errors were rated by 0 (no compensatory errors) or 1 (presence of compensatory errors) [27].

**Fig. 3:**
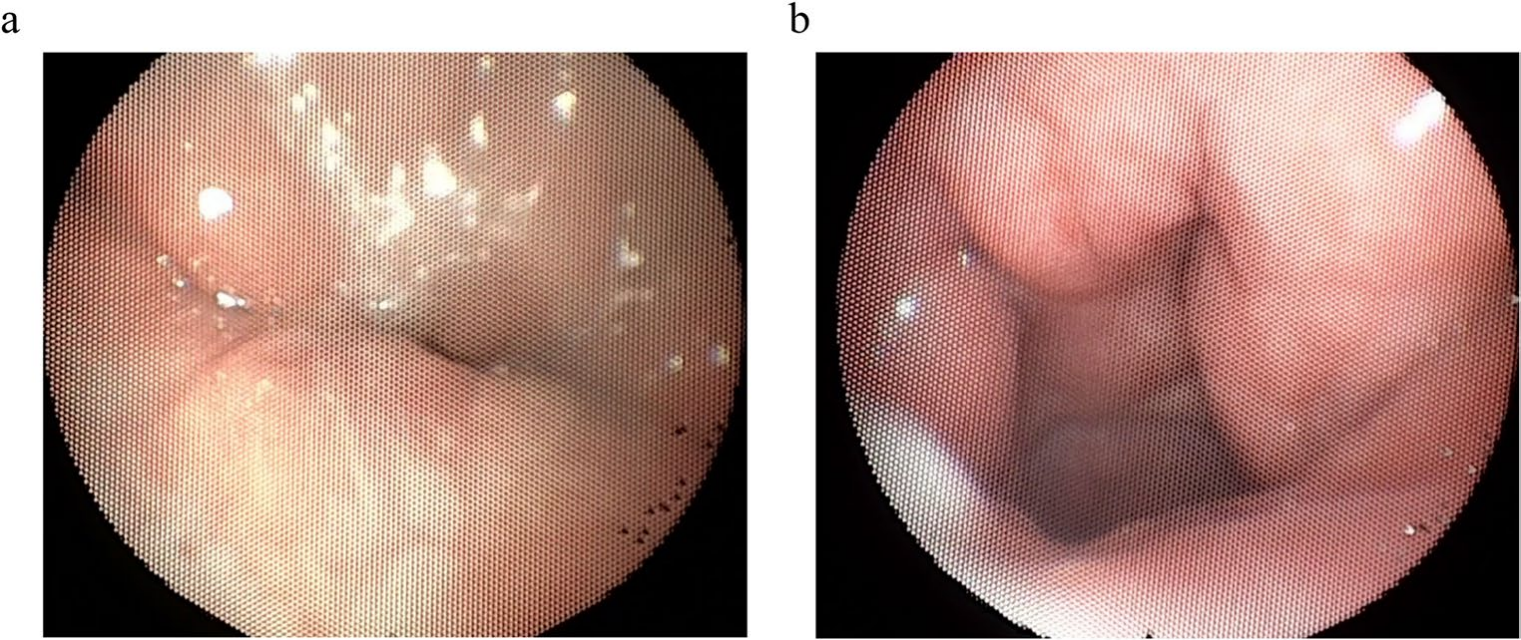
**a** Showing complete closure of the VP valve after cleft palate repair by primary FPBF. **b** Showing incomplete closure of the VP valve after cleft palate repair by primary TFP

Nasopharyngoscopic examination: (Fig:3a,b)

The used nasopharyngoscope is a flexible nasal endoscope, 3.4 mm in diameter with a camera (Henke Sass Wolf- Gmbh Nasolaryngoscope) that is connected to a computer. It was used to evaluate the degree of velopharyngeal valve competence during speech by rating the degree of VP closure. It was completed by asking the child to repeat various syllables, words, phrases, and numbers (1 to 10). The VP valve closure was rated on a 5-point scale(0–4) as (0) reflected that the valve is completely open, while (4) reflected that the valve is completely closed [28].

#### Statistical methods

The data produced from the four assessment methods were handled using Excel sheets. Mean and standard deviation were calculated for each assessment method in the FPBF group and in the TFP. *Kolmogorov-Smirnov* test was used for data normality assessment. The readings produced from both surgical techniques were compared using a paired t-test for each of the 4 speech assessment methods. The level of significance was maintained at 0.05. The power of study was set at 0.8.

## Results

The data of all the included 30 patients were analyzed. The baseline characteristics are mentioned in Table 1.

**Table 1:** Baseline characteristics of the included patients

		FPBF	TFP
Cleft Laterality	Right	7	5
	Left	8	10
Age		4.23±0.79	4.4±0.73
Gender	Male	9	5
	Female	6	10
Associated Anomalies		0	0

FPBF: furlow palatoplasty with buccal myomucosal flap; TFP: two flap palatoplasty

For data normality, all data was normally distributed. A statistically significant improvements were detected in the 4 assessment methods (amount of hypernasality, speech intelligibility, compensatory misarticulation and nasopharyngoscopy) while comparing the FPBF group’s scores to the TFP group’s scores as shown in Tables 2 and 3.

**Table 2:** Evalution of hypernasality, speech intelligibilty and compensatory misarticulation between the 2 groups compared using the *independent t-test*

Hypernasality								Speech Intelligibility								Compensatory Misarticulation							
FPBF		TFP		Diff			*P*-value	FPBF		TFP		Diff			*P*-value	FPBF		TFP		Diff			*P*-value
Mean	SD	Mean	SD	Mean diff	CI 95%	SD		Mean	SD	Mean	SD	Mean diff	CI 95%	SD		Mean	SD	Mean	SD	Mean diff	CI 95%	SD	
0.13	0.19	1.3	0.26	−1.2	−1.34 to −0.99	0.32	0.0001*	0.13	0.19	1.3	0.26	−1.2	−1.34 to −0.99	0.32	0.0001*	0.13	0.19	0.73	0.25	−0.6	−0.76 to −0.43	0.30	0.0004*

FPBF: furlow palatoplasty with buccal myomucosal flap; TFP: two flap palatoplasty;*: significant (*p* < 0.05); SD: standard deviation; CI: Confidence interval

**Table 3:** Evalution of Nasopharyngoscopy between the 2 groups compared using the*independent t-test*

Nasopharyngoscopy							
FPBF		TFP		Diff			
Mean	SD	Mean	SD	Mean diff	CI 95%	SD	P-value
3.86	0.19	2.8	0.22	1.066	0.90 to 1.21	0.28	0.0001*

*TFP* two flap palatoplasty, *FPBF* furlow palatoplasty with buccal myomucosal flap, *SD* standard deviation, *CI* confidence interval

## Discussion

Velopharyngeal insufficiency is the most frequent complication associated with primary palatoplasty [6]. Some patients with repaired cleft palate have a VPI asthe soft palate fails to contact the pharyngeal wall properly [7, 8].

Bangun et al. [29] documented that the age of the patient at the time of surgical repair and the surgical technique are the most influential factors on speech outcome. Early repair of cleft palate improves the speech outcome but it associates with growth retardation. All cleft palate were primarily repaired in this study between 9 and 12 months. It is considered the most suitable age to improve speech development and avoid growth retardation [30, 31]. 

Regarding the surgical technique, TFP was the most preferred technique in the past and for a long time. It is a simple technique that dose not require an expert surgeon [16]. Patients who were treated with TFP experienced a low rate of fistula [17]. However, the length of the repaired soft palate is inadequate. In addition, it associates with a high incidence of VPI as the resulted scar at the relaxing incision site tend to contract, impaired the function of the palatini muscles, and impaired the contact between the soft palate and pharyngeal wall [17, 18]. Because of the drawbacks of TFP, a paradigm shift has occurred to the surgeons’ decision-making process toward using FPBF. Although FPBF is a technique that needs an expert surgeon, the treated patients have adequate VP function. It provides a proper reorientation of the palatini muscles, lengthen the soft palate with the Z-plasty design and buccal myomucosal flap that substitutes the palatal deficient tissue and allows the layers closure without tension regardless to the cleft width. In addition, the resulted scar at the relaxing incision site of TFP with its complications were avoided [13, 22].

The study’s null hypothesis stated that a primary FPBF had a good speech outcome and VP function rather than TFP. Accordingly, the plan of this study was to evaluate the speech outcome of primary Furlow with buccal myomucosal flap versus two flap palatoplasty in patients with cleft palate. 

The speech assessment in this study was performed between the age of 4 and 8 years as the child become more cooperative during the perceptual assessment around 3 years and was able to tolerate the nasopharyngeoscope procedure around the age of 4 years. In addition, the preferred age for VPI management is around 4 years, as the period of speech development is considered [10, 31–34].

Perceptual assessment evaluates the VP function by rating the hypernasality, compensatory Misarticulation and Speech Intelligibility. While the nasopharyngeoscope visualizes and evaluates the VP closure [35, 36]. 

In this study, statistically significant improvements were detected in all assessment methods (amount of hypernasality, speech intelligibility, compensatory misarticulation and nasopharyngoscopy VP valve competence) while comparing the FPBF group’s scores to the TFP group’s scores. These findings followed the previous studies by Mann et al. [37] in 2011 and Elrouby et al. [14] in 2025, whose found that the patients treated with FPBF showed better VP function and speech development. This finding may be attributed to the FPBF that provides a proper levator muscle reorientation, and sufficient soft palate length with the Z-plasty design and buccal myomucosal flap that augments the palatal deficient tissue and allows the layers closure without tension regardless to the cleft width. In addition, the resulted scar at the relaxing incision site of TFP with its complications were avoided [13, 22].

This study was the first one that compared the effect of primary FPBF versus primary TFP on speech development with a pre-calculated sample size, and this was considered the main points of study strength. However, this study design was categorized as a cohort study that is susceptible to selection biases, and it introduced a limitation of this study. A further limitation was the inability to blind the speech pathologist to the surgical technique during the intraoral examination, as the healed palate often reveals features indicative of the repair type. In addition, a randomized controlled trial (RCT) design was not feasible for this specific research question. Palatoplasty, the primary surgical intervention, is a time-sensitive, standard-of-care procedure that must be performed in infancy (9–11 months) to ensure normal feeding and auditory tube function and to provide the anatomical basis for speech development. Furthermore, the outcome of interest (speech proficiency) cannot be reliably assessed until the child is old enough to produce complex sounds, typically at 3–4 years of age. This inherent and unavoidable delay between intervention and outcome measurement creates an insurmountable barrier for a traditional RCT in this clinical context.

Further studies are recommended to compare the primary furlow palatoplasty with versus without buccal myomucosal flap. In addition, a further longitudinal follow-up study will be needed to evaluate the sustainability of these results into adolescence, as this study demonstrates the positive outcomes in early childhood.

## Conclusions

Based on the followed methodology and the obtained results, primary FPBF might be able to improve the amount of hypernasality, speech intelligibility, nasopharyngoscopy VP valve, and compensatory misarticulation in comparison to TFP in patients with cleft palate. Accordingly, it might be the surgical palatal repair technique of choice in patients with cleft palate.

## Supplementary Information

Below is the link to the electronic supplementary material.

Supplementary Material 1 (MP4 10.6 MB)

Supplementary Material 2 (MP4 13.3 MB)

## Data Availability

The corresponding author is responsible about all the study’s data that available upon request.

## Abbreviations

FPBF: Furlow palatoplasty with buccal myomucosal flap
STROBE: Strengthening the Reporting of Observational studies in Epidemiology
TFP: Two flap palatoplasty
VP: Velopharyngeal
VPI: Velopharyngeal insufficiency
